# CT-Guided Percutaneous Transthoracic Needle Biopsies Using 10G Large-Core Needles: Initial Experience

**DOI:** 10.1007/s00270-015-1098-z

**Published:** 2015-05-13

**Authors:** Ulrich C. Lalji, Joachim E. Wildberger, Axel Zur Hausen, Matyas Bendek, Anne-Marie C. Dingemans, Monique Hochstenbag, Marco Das

**Affiliations:** Department of Radiology, Maastricht University Medical Center, P. Debyelaan 25, PO Box 5800, 6202 AZ Maastricht, The Netherlands; GROW School of Oncology and Developmental Biology, Maastricht University Medical Center, P. Debyelaan 25, PO Box 5800, 6202 Z Maastricht, The Netherlands; Department of Pathology, Maastricht University Medical Center, P. Debyelaan 25, PO Box 5800, 6202 AZ Maastricht, The Netherlands; Department of Pulmonology, Maastricht University Medical Center, P. Debyelaan 25, PO Box 5800, 6202 AZ Maastricht, The Netherlands

**Keywords:** Image-guided biopsy, Large-core needle, Biopsy, Computed tomography (CT), Interventional radiology, Lung neoplasms, Pneumothorax

## Abstract

**Purpose:**

Using large-core biopsy needles in CT-guided percutaneous transthoracic needle biopsies (PTNB) may be advantageous in terms of larger specimens, which facilitate more extensive histopathological, immunohistochemical, and molecular examination of tumor tissue. The aim of this study was to evaluate the success rate and safety in CT-guided PTNB using 10G large-core biopsy needles.

**Methods and Materials:**

35 patients with intrathoracic lesions suspected of malignancy underwent CT-guided PTNB using dedicated large-core biopsy needles (10G Spirotome™, Medinvents, Hasselt, Belgium). Location, tumor size, number of pleural passes, number of biopsies, histologic result, and complications (pneumothorax, bleeding) were recorded.

**Results:**

Lesion location varied from pleural to hilar location. Mean tumor size was 3.5 cm (range 0.7–9.2 cm). Only one pleural passage was necessary in all patients. Mean distance from the pleura to the lesion was 2.6 cm (max 9.2 cm). Large-core biopsy (10G) was successful in 88.6 %. Pneumothorax was found in 40 %. Minor intraparenchymal bleeding was present in 14 patients. No major complications were recorded.

**Conclusion:**

Large-core biopsy with 10G did not show higher complication rates compared to literature. It is technically feasible and safe. The obtained larger specimens may especially be helpful for the increasing demands of extensive molecular analysis for stratified patient care.

## Introduction

Over recent years, the treatment of lung cancer has changed significantly, necessitating a more complex approach to pathological diagnosis of individual lung tumor specimens. In addition to the precise histopathological diagnosis and subtyping of lung tumors—which might necessitate the use of supportive immunohistochemistry—the detection of possible genetic mutations, translocations, and amplifications has gained more and more importance. Molecular analysis of driver mutations is standard care in lung cancer in the era of personalized treatment as the presence of a driver mutation can have prognostic and predictive value-targeted therapy [1, 2].

Only few of these molecular alterations can be detected by immunohistochemistry such as v-raf murine sarcoma viral oncogene homolog B1 (BRAF) and anaplastic lymphoma kinase (ALK) (mutations cannot be detected by immunohistochemistry but the altered protein might be detected if an antibody is available against the altered protein, e.g., BRAF). But more often, sophisticated molecular pathological methods like chromogenic or fluorescent in situ hybridization and sequencing have to be applied. Both, the most common, for example epidermal growth factor receptor (EGFR), Kirsten rat sarcoma viral oncogene homolog (KRAS) and the less frequently occurring [e.g., ALK, c-ros oncogene 1 (ROS1), rearranged during transfection (RET), v-erb-b2 erythroblastic leukemia viral oncogene homolog 2 (HER2)] [3, 4] mutations are considered as yet to be mutually exclusive, which means that in some cases subsequent tests may be needed to detect or to exclude a given mutation. Furthermore, in clinical studies, with new targeted drugs more and more tumor tissue is required for central biomarker testing in order to select which patients will benefit from the investigated compound or regimen [5]. Since at least 200–400 malignant cells in a biopsy specimen are already needed to obtain a histological diagnosis [6–8], the additional expanding panel of immunohistochemical and molecular analysis requires more tumor tissue and this may result in more biopsies or repeated biopsy when standard size needles are used.

CT-guided percutaneous transthoracic needle biopsy (PTNB) is widely accepted as an accurate diagnostic method to obtain tissue for histological diagnosis in lung lesions [9, 10] 77–96 % and low rates of complications requiring treatment [9, 11–18]. Compared to cytological sampling methods such as bronchoalveolar lavage or fine needle aspiration biopsy (FNAB), histological sampling leads to higher diagnostic accuracy and also allows evaluation of tumur architecture [8]. As described by Dacic, the mean number of cells obtained by using 21G needles is ≥100, while with 18G CT-guided PTNB the number of cells is ≥500 [6]. The mean number of cells obtained by using different biopsy methods is summarized in Table 1.

**Table 1:** Number of cells per biopsy—adapted from Pirker et al. [8]

Technique and needles	Number of cells	Number of biopsies
21-g needle aspiration	≥100	4
19-g needle aspiration	≥150	4
Transbronchial biopsy 21-g	≥300	4
CT-guided needle biopsy	≥500	2–3

The use of a large-core 10G needle in CT-guided PTNB may therefore be advantageous as with a maximal diameter of 3 mm its maximal calculated volume is nearly nine times the volume of a 18G needle (maximal diameter 1 mm), with consecutively a potential ninefold increase in the number of cells per specimen. Moreover, as the diameter of the larger core is about three times that of the 18G core, more tissue sections can be made for immunohistochemical analysis, which means that one single core biopsy might already be enough for all necessary tests (Table 2).

**Table 2:** Lesion characteristics, procedure properties, and histological outcome per patient

	Age (years)	Size (cm)	Distance to lesion (cm)	Location of lesion	Procedure time (min)^a^	Patient position	Histology result
1	72	2.3	9.1	rll	28	Prone	Adenosquamous carcinoma
2	63	3.6	3.2	lll	23	Prone	Adenocarcinoma
3	68	2.5	1.7	lul	31	Supine	Adenocarcinoma
4	56	3.3	5.5	rll	49	Prone	Adenocarcinoma
5	76	2.6	1.4	lul	30	Supine	Adenosquamous carcinoma
6	69	1.3	4.4	lll	55	Prone	Amyloid
7	38	5.7	3	rll	45	Prone	Infection
8	65	0.9	3.1	rll	23	Prone	Adenocarcinoma
9	74	3.2	1.4	rll	20	Prone	Adenocarcinoma
10	67	0.8	1.2	rll	27	Prone	Anthrasilicosis
11	73	1.2	5.3	rul	43	Left side	Adenocarcinoma
12	69	2	3.9	rll	27	Prone	Adenocarcinoma
13	66	3.1	2.8	rml	32	Supine	Adenocarcinoma
14	68	7.6	5.5	rul	26	Supine	Sarcomatoid carcinoma
15	57	6.1	0	Pleural	36	Prone	Schwannoma
16	70	2.7	2,5	rul	53	Left side	NSCLC, NOS
17	71	1.8	0	rll	44	Left side	Squamous metaplasia
18	76	3.9	3.6	rul	32	Supine	SCLC
19	65	0.7	2.5	rul	49	Supine	Endometrial carcinoma^c^
20	60	1.5	6.3	rul	33	Prone	NSCLC
21	69	5.8	2.1	rul	32	Supine	Necrosis
22	45	4.1	0.2	lll	37	Prone	Squamous cell carcinoma
23	76	3.9	0	rul	28	Prone	Squamous cell carcinoma
24	71	3.9	0	rll	28	Left side	Squamous cell carcinoma
25	68	7.1	0	rul	28	Prone	Inflammation
26	58	4.3	0	rll	40	Left side	Rectal carcinoma^c^
27	75	1.9	3.6	rul	33	Supine	Chronic inflammatory changes
28	69	6.7	3.5	rul	26	Prone	Adenocarcinoma
29	48	1.4	2.1	lll	45	Prone	Adenoid cystic carcinoma
30	49	3.7	0.7	rll	30	Left side	Adenocarcinoma
31	62	5.4	0.6	lll	20	Prone	Adenocarcinoma
32	58	2.8	7.1	rul	35	Prone	Adenocarcinoma
33	79	3	0	Cavity^b^	50	Supine	Adenocarcinoma
34	53	4.3	1.9	rul	45	Supine	Adenocarcinoma
35	73	6.1	2.9	rul	27	Prone	Squamous cell carcinoma
Mean	65	3.5	2.6		34.6		

*rul* right upper lobe, *rml* right middle lobe, *rll* right lower lobe, *lul* left upper lobe, *lll* left lower lobe, *NSCLC* non-small cell lung carcinoma, *SCLC* small cell lung carcinoma, *NOS* not-otherwise specified

^a^Total procedure time: in room time

^b^Lesion in post pneumonectomy cavity

^c^Lung metastasis

Therefore, the aim of this retrospective analysis was to evaluate the success rate and safety in CT-guided PTNB using 10G large-core biopsy needles.

## Methods and Materials

**Fig. 1:**
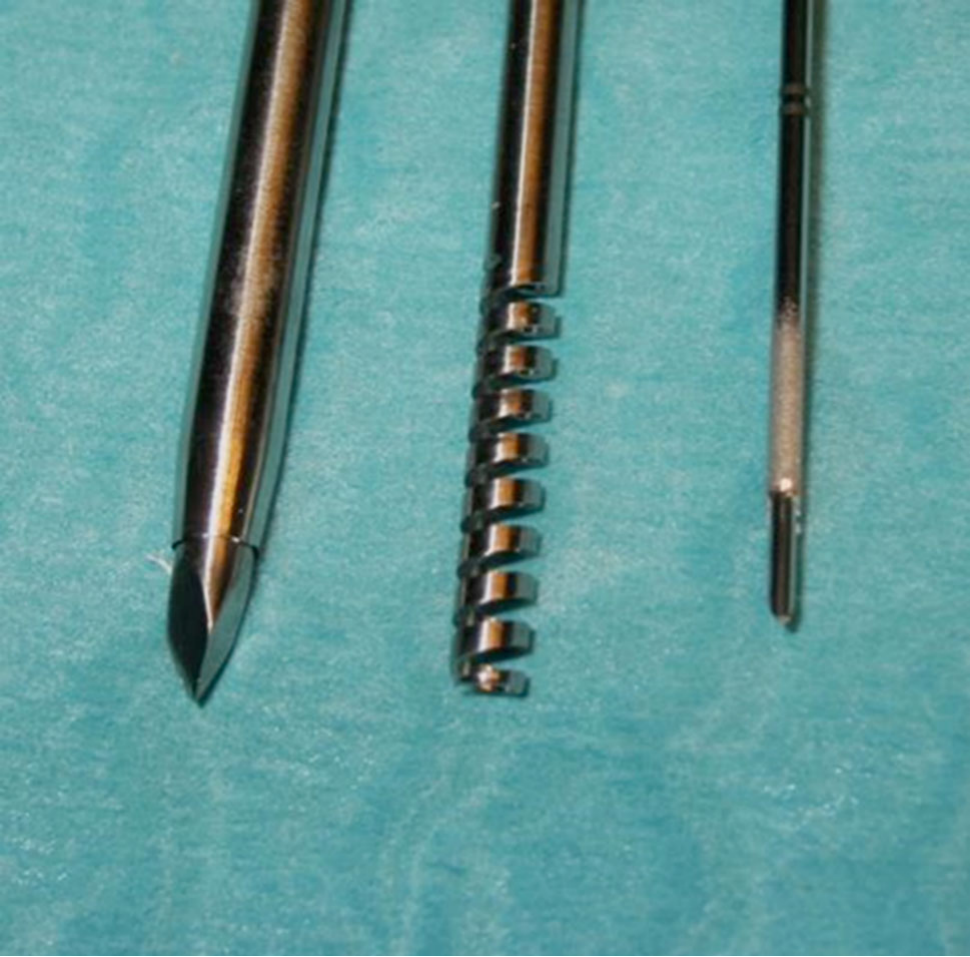
10G biopsy needle (*middle*) with coaxial needle system (*left*) and in comparison to 18G biopsy needle (*right*). Notice that in the 10G system the coaxial needle also serves as cutting needle

In the period of December 2011 till January 2013, a total of 35 patients (17 male, 18 female, mean age 65 ± 9.5 years) with suspected intrathoracic malignancy underwent CT-guided PTNB under local anesthesia using dedicated l0G large-core biopsy needles (Spirotome™, Medinvents, Hasselt, Belgium) (Fig. 1). Patients were referred to our department either as part of the routine evaluation of a intrathoracic mass or as part of a second opinion after biopsies elsewhere did not provide a definitive diagnosis. The biopsies were performed under CT-guidance (SOMATOM Sensation 16, Siemens Healthcare, Forchheim, Germany) using breath-hold technique if necessary. Biopsies with 10G were only performed by or under the supervision of two radiologists with 9 and 6 years of experience regarding transthoracic biopsies. The need for informed consent was waived by the local ethics committee (METC 14-4-021).

### Biopsy Protocol

All patients were referred to the radiology department in order to obtain material for further histopathological analyses of intrapulmonary lesions. In all patients, at least one diagnostic CT scan of the chest was performed in routine workup. If a positron emission tomography-scan (PET-scan) was performed in the workup, both the diagnostic scan and the PET-scan were used to plan the biopsy, but no other imaging technique other than CT was used for biopsy guidance. A helical non-contrast CT scan was performed in all patients as part of the biopsy protocol to confirm size and location directly before the procedure. If necessary, iodinated contrast media was administered for better delineation of vascular structures. After localization on helical CT scan, the final needle path was planned and local anesthetics were administered along the planned route. The 10G biopsy system consists of a coaxial needle system and the biopsy needle. Figure 1 demonstrates the size difference of the 10G needle in comparison to a standard 18G (Biopince^®^ Full-Core Biopsy Instruments, Angiotech, PBN Medicals, Stenlose, Denmark) biopsy needle. Once the coaxial needle was placed close to or in the lesion, the biopsy needle was inserted and then screwed into the lesion. The coaxial needle was then used as a cutting needle in order to retrieve the specimen. In general, fluoroscopy is not used in our Department in PTNB. Obtained tissue samples are fixated immediately in a formaldehyde solution.

Immediately after biopsy, the patients underwent a low-dose helical non-contrast CT scan to assess procedure related complications. Patients were observed for 2 h after procedure. After this period, a chest X-ray was routinely made to check for complications. When no complications were observed the patients were discharged. In case of a complication, the need for treatment and prolonged hospital stay was discussed with the chest physician. When an asymptomatic pneumothorax was observed, the decision to place a chest tube or discharge the patient was made by the chest physician.

Tumor locations, tumor size, number of pleural passes, number of biopsies, procedure time (total in room time), complications (pneumothorax, bleeding, and hemoptysis) were recorded. Complications were graded as minor or major according to the quality improvement guidelines as stated by the ACRl [19]. Diagnostic performance was recorded by comparing histological outcome with definitive pathology at resection when possible or, if no resection was performed by looking at clinical outcome. Descriptive statistics were used.

## Results

Location of the lesions varied from pleural to hilar location with mean pleura to lesion length being 2.6 cm (range 0–9.2 ± 2.2 cm). Mean tumor size was 3.5 cm (range 0.7–9.2 ± 1.8 cm). Only one pleural passage was necessary in all patients. In 6 patients (17.1 %), 2 biopsy specimens were taken during the procedure without the need for a second pleural passage. In 1 case (2.8 %), 3 biopsies were taken with one pleural passage. In the remaining 28 cases (80.1 %), only one biopsy was taken.

**Fig. 2:**
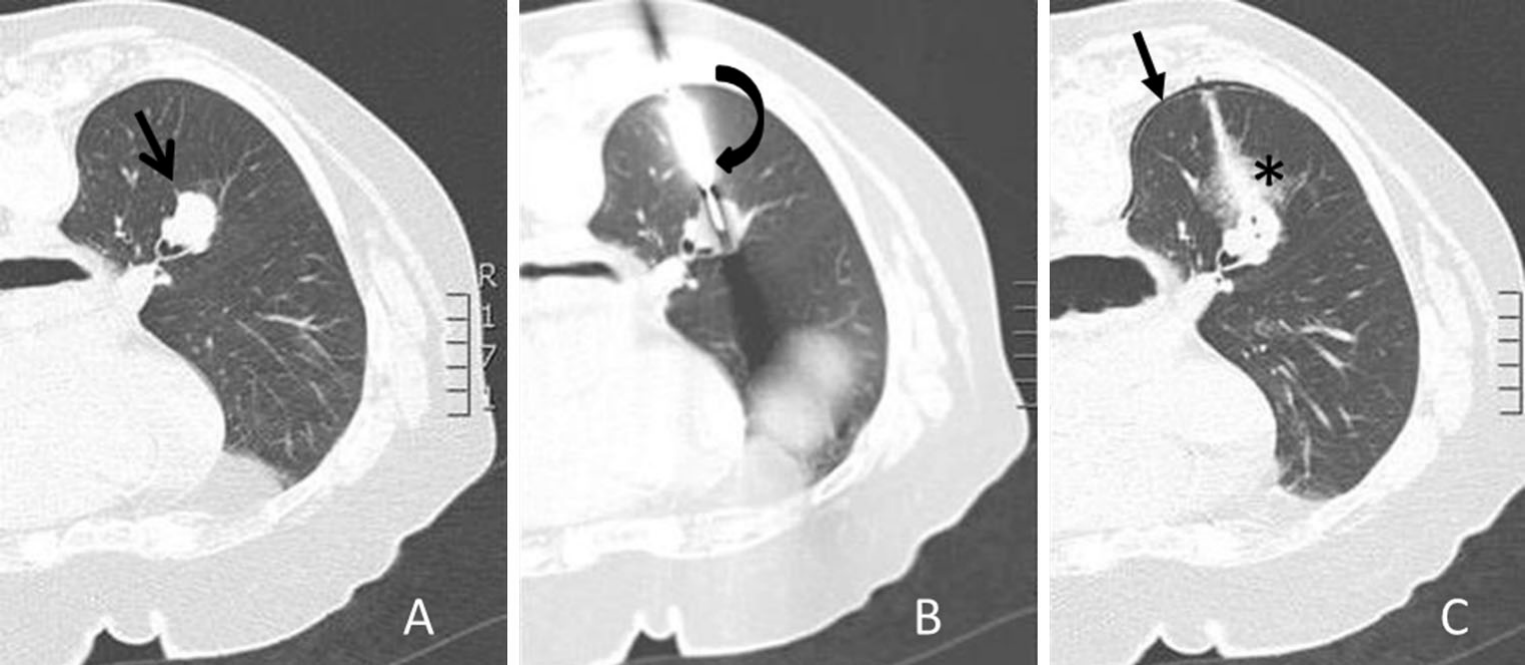
**A** Example of centrally located lesion (*black arrow*). **B** Biopsy needle inside lesion (*curved black arrow*). **C** Post-procedural image reveals needle tract bleeding surrounded by a minor intraparenchymal bleeding (*asterisk*) and small pneumothorax (*small arrow*), all of which were not clinically relevant

**Fig. 3:**
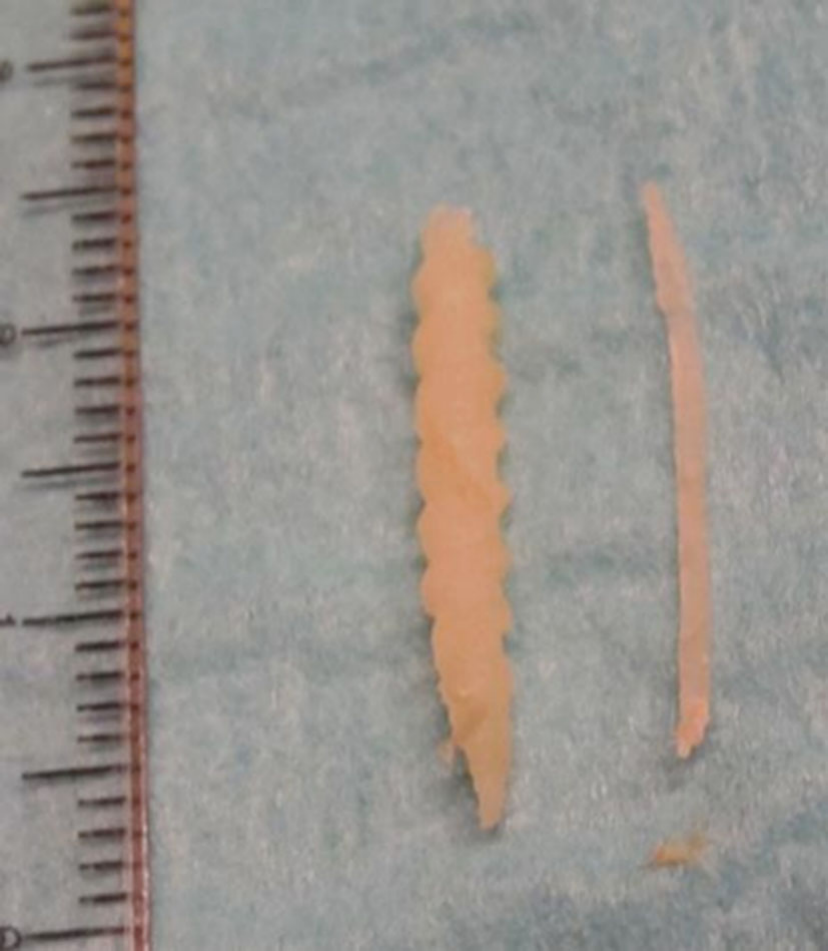
Comparison of artificial tissue specimen taken by 10G needle (*left*) and 18G needle (*right*)

Figure 2 demonstrates an example of a CT-guided biopsy taken with a 10G needle. Figure 3 demonstrates biopsy specimens taken by 10G needle in comparison to 18G needle biopsy from two different patients. Mean procedure time (in room time) was 34.6 min (range 20–55 min).

On the helical thin slice CT performed, immediately after the biopsy needle was removed a pneumothorax was observed in 40 % (14 of 35 patients). None of these patients required placement of a chest tube at that time. The post-procedural chest X-ray 2 h later showed a pneumothorax in 20 % (7 of 35) of patients

In 2 patients without symptoms (5.6 %) a chest tube was placed by order of the pulmonologist because of the size of the pneumothorax, resulting in a prolonged hospital stay of respectively 4 and 5 days therefore registered as a major complication.

No hemoptysis or hematothorax was seen. Minor intraparenchymal bleeding was present in 16 cases (45.7 %) on the helical CT scan directly after the procedure, which did not result in hemoptysis or further complications. No late complications were observed. An overview of the observed complications is given in Table 3.

**Table 3:** Complication rate of 10G biopsies (*n* = 35)

Minor complications	Major complications
Pneumothorax On CT 14 (40 %) On X-ray 7 (20 %)	Chest tube placement requiring prolonged hospital stay 2 (5.7 %)
Parenchymal bleeding 16 (45.7 %)	

The large-core biopsy resulted in definitive histological diagnosis in 31 of 35 patients. In 4 patients, histopathological analysis was not informative with regard to the presence of tumor (detailed information on summary findings and histology results is given in Table 2).

## Discussion

Treatment of lung cancer has moved towards individualized treatment using the combined assessment of immunohistochemical markers, mutational analyses, and FISH techniques in order to assess the exact histology and to detect, for example, KRAS and EGFR mutations or ALK translocations [6, 8, 20]. These assessments increased the need for adequate and large tumor samples. As diverse biopsy techniques yield different amounts of tissues, it is not always possible to apply the complete spectrum of tests to the available tissue, sometimes requiring repeat biopsies [6]. In our study, immunohistochemistry analyses were performed in 33 of 35 patients and molecular analysis was performed in 12 cases.

**Fig. 4:**
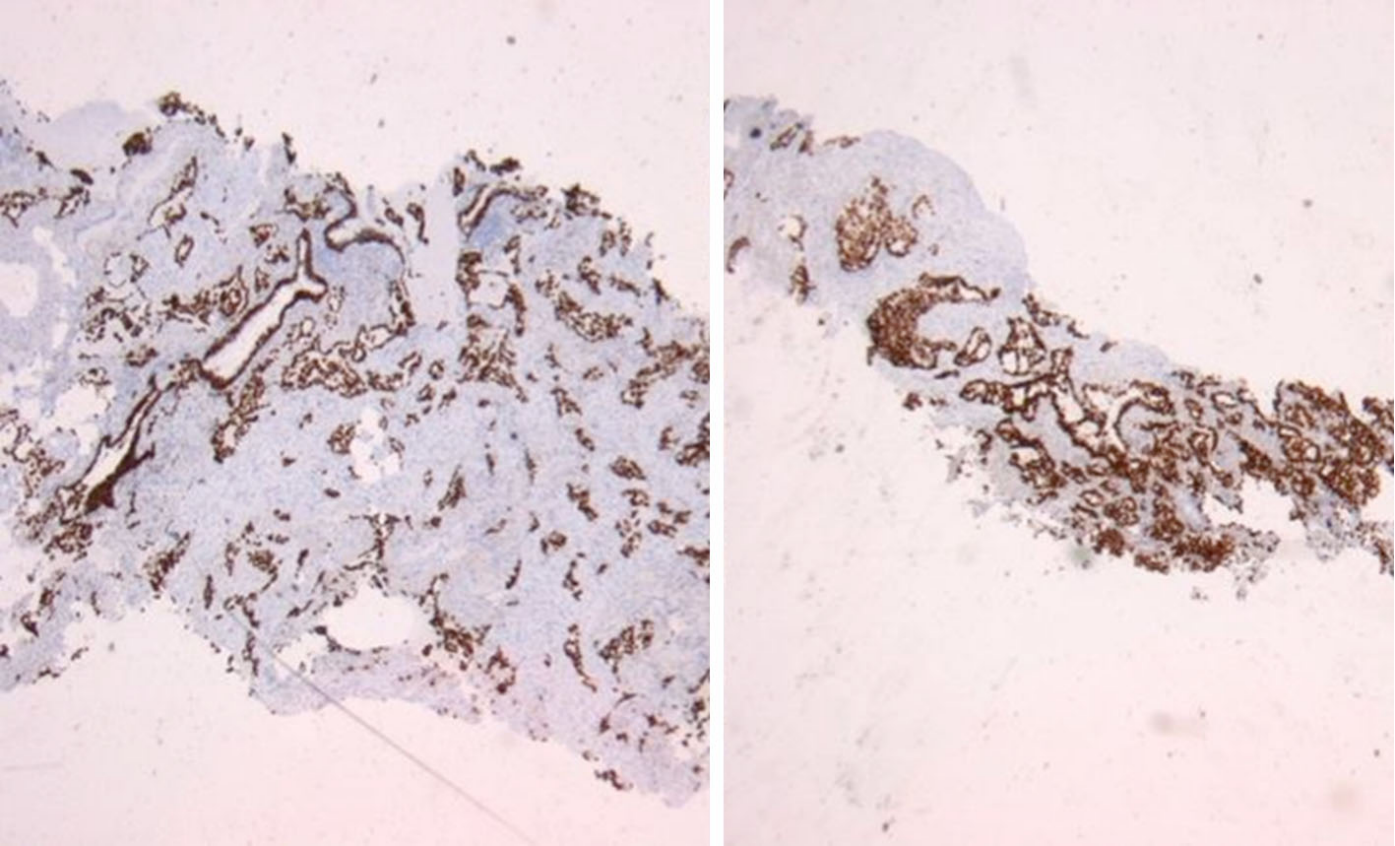
Comparison of core samples: 10G (*left*) historical workup and 18G (*right*) historical workup. TTFE staining light microscopy ×10 magnification

Large-core (10G) biopsy needles might improve diagnostic accuracy since their potential ninefold increase in tissue volume compared to traditional 18G needles allows more extensive histological analysis leaving enough material to perform all molecular tests. Figure 4 shows a comparison of a histological image of core samples taken by 10 G and 18 G needles. To our knowledge, this is the first report on the use of 10G biopsy needles in PTNB.

In 6 cases, a second specimen was taken during the procedure. In 1 case, the second biopsy was taken for microbiological analyses as is customary. In 2 cases, a second biopsy specimen was taken after the first specimen only showed disintegrating tissue at visual inspection. In these 2 cases, unfortunately even the second biopsy specimen did not result in a definitive histological diagnosis. In the remaining 3 cases, a second biopsy was taken as is customary in our institution when using 18G needles. In hindsight, the second biopsy was not necessary since the first obtained specimens were of sufficient quality. Therefore, the standard procedure was adapted to ensure only one biopsy should be taken if on visual inspection the specimens were deemed to be of sufficient quality. In one case, 3 biopsies were taken because of disintegrating specimens at visual inspection which eventually only showed necrotic tissue combined with active inflammation. In hindsight, the targeted area for biopsy was a post-obstructive atelectasis which was cleared on later follow-up scans. The more centrally located cause of the obstruction was not targeted at biopsy. In the remaining 28 cases, only one biopsy was needed of which one was not conclusive regarding to the histopathological diagnosis. In our institute, a cytopathologist is generally not present during biopsy procedures.

Of the 4 cases, in which histopathological analysis did not reveal the presence of malignancy, 3 showed inflammatory changes and 1 case of necrosis with acute inflammatory changes. It is doubtful that an 18G biopsy needle would have had a better result. In the follow-up of these cases, one patient died of other causes 1 year after the biopsy without a definitive diagnosis. The second patient developed a skin metastasis which turned out to be a squamous cell carcinoma of probable lung origin. The third patient was treated with radiotherapy in the assumption of a lung malignancy and has stable disease. The fourth patient underwent a second biopsy with 10G which resulted in the diagnosis of a squamous cell carcinoma. Even though 10G resulted in a definitive diagnosis, the first biopsy yielded a negative result. Therefore, we did not include the second biopsy in our results.

Several reports show that the use of 18–22G needles for a CT-guided PTNB is a safe method with a relatively low complication rate. Choo et al. reported initial results on 18G needle biopsy with cone beam CT (CBCT) guidance and reported a sensitivity for nodules <1 cm with 96.7 %, specificity with 100 % and accuracy of 98 % with a complication rate of 13 % (6.5 % pneumothorax rate, 5.6 % hemoptysis) [12]. Beslic et al. even compared 14G biopsies with 20–22G FNABs and concluded that core biopsies yielded a higher percentage of representative samples than FNABs (97 vs. 80 %) [21]. Some authors suggest higher complication rates for larger needles, but other authors demonstrated even lower complication rates with large-core biopsies. For example, Anderson et al. found a pneumothorax rate of 35.1 % for FNAB and 15.9 % for 18G needle biopsies [22]. Ko et al. reported on factors which influence the pneumothorax rate [23]. Overall pneumothorax rate was 39 %, while emphysema and lesions along fissures were associated with higher risk of pneumothorax. Furthermore, Nakamura et al. reported in their patient population, patient age, presence of emphysema, lesion size, needle path length, number of pleural passages, as well as the smallest angle between the pleura and the needle as significant factors for the development of pneumothorax (which was 59 %) [24, 25]. The number of pneumothorax in our series seems relatively (40 %) high using 10G needles. However, all patients with a pneumothorax were asymptomatic. This rate observed on the CT scan performed immediately after withdrawal of the needle was lower on chest X-ray. There is no clear consensus on observation and follow-up of patients after biopsy. In our institution, all patients undergo a low-dose thin slice helical non-contrast CT immediately after the biopsy needle has been removed as part of the biopsy protocol. Any small amount of air visualized in the pleural space was considered a pneumothorax. Since there seems to be no consensus in the grading of pneumothorax on CT or chest X-ray, we choose to evaluate the need for chest tube placement on symptoms (shortness of breath, chest pain, etc.) or estimated increase of pneumothorax rather than estimated pneumothorax size alone. Furthermore, all patients underwent a chest X-ray two hours after biopsy to detect complications. A retrospective analysis of 120 consecutive patients biopsied with 18G needles in our institute showed a diagnostic accuracy of 87 % and pneumothorax rate of 45 %. It is not clear from the published reports whether all institutes followed the same regimen. In addition, no bleeding complications were observed and prolonged hospital stay was observed in 2 of the total 35 patients due to chest tube placement. Even though both patients were asymptomatic, according to the quality improvement guidelines from the ACR [19], this must be classified as a major complication. One of these patients with a prolonged stay of 4 days was already planned a surgery of a head and neck tumor which took place on day 3 of his 4-day stay.

### Limitations

We are presenting our initial results on 10G PTNB of pulmonary lesions. Therefore, there are certain limitations.

First, the number of patients is limited. A more extensive evaluation has to follow, especially, in terms of comparing the amount of tissue in the obtained specimen and its value for extensive histological analysis in lung cancer.

Second, biopsies with 10G were only performed by or under the supervision of two radiologists. If not present, the needle choice for other biopsies was to the discretion of the performing radiologist. This of course leaves some bias in the patient selection.

## Conclusion

Large-core biopsy of lung lesions with 10G needles is feasible and safe with comparable complication rates as in case of standard 18G biopsies. Therefore, we conclude that large-core needles are a safe method in obtaining larger tissue specimens in pulmonary lesions offering the possibility to assess the complete spectrum of the continuously growing number and needs of histopathological, immunohistochemical, and molecular testing in lung cancer in order to guarantee best patient care.
